# The Use of Modal Analysis in Addition Percentage Differentiation, and Mechanical Properties of Ordinary Concretes with the Addition of Fly Ash from Sewage Sludge

**DOI:** 10.3390/ma14175039

**Published:** 2021-09-03

**Authors:** Gabriela Rutkowska, Mariusz Żółtowski, Michał Liss

**Affiliations:** 1 Institute of Civil Engineering, Warsaw University of Life Sciences (SGGW), 02-776 Warsaw, Poland; 2 Water Centrum, Warsaw University of Life Sciences (SGGW), 02-776 Warsaw, Poland; mariusz_zoltowski@sggw.edu.pl; 3 Faculty of Mechanical Engineering, UTP-University of Science and Technology in Bydgoszcz, 85-796 Bydgoszcz, Poland; michal.liss@utp.edu.pl

**Keywords:** concrete, sewage sludge, fly ash, cement, vibrations, modal analysis

## Abstract

Production cost reduction and constraints on natural resources cause the use of waste materials as substitutes of traditional raw materials to become increasingly important. The dynamic development of sewerage systems and sewage treatment plants leads to increases in the produced sewage sludge. According to the Waste Law, municipal sewage sludge can be used if it is properly stabilized. This process results in significant quantities of fly ash that must be utilized. This paper presents investigation results of partial cement replacement influence by the fly ash from sewage sludge on concrete parameters. The results confirm the possibility of fly ash waste applications as a cement substitute in concrete manufacturing. In the later parts of the publication, a pilot study was conducted using the modal analysis methodology and aimed at checking the hypothesis of whether vibration methods can be used in the assessment of the amount of the admixture used in concrete and the effect it has on concrete properties. This is the first time that vibration tests have been used to determine the diversity of the concrete mix composition and to distinguish the percentage of ash added. There are no studies using modal analysis to distinguish the composition of a concrete mix in the scientific literature. The article shows that the vibration test results show the differentiation of concrete composition and can be further improved as a method for determining the composition of mixtures and for distinguishing their mechanical properties. These are only pilot studies, which, in order to develop the target cognitive inference, should be performed in the future on a significantly enlarged number of the studied samples.

## 1. Introduction

Concrete, sometimes referred to as the stone of the present day, is by far the most widely used composite material among man-made materials, second only to water in the entire complex of used materials and without which modern construction would not function. It is an ecological composite that is often made of local raw materials—aggregate (as a filler), cement (as a binder), water, admixtures, and possibly mineral additives. It is a safe product that guarantees the stability and load-bearing capacity of a structure.

One of the most important issues in the development of the construction sector is the continued efforts to make concrete an ecological, even more environmentally friendly material for the environment. The search is undoubtedly indispensable in the case of developing the composition of a concrete mix, the two components of which—aggregate and cement—contribute to the anthropopressive effects at their production and acquisition stages. The big problem is still the fact that during the production of 1 ton of cement, 0.5 to 1 ton of greenhouse gases are produced, which, according to various data, constitutes 6–8% of the total anthropogenic emission. Research shows that the production and processing of this binder is responsible for 85% of the CO_2_ production in the entire concrete industry. To reduce the environmental impact of the produced concrete, its two main components, aggregate and cement, can be replaced by more sustainable alternatives. Natural aggregate can be replaced with recycled aggregate, while Portland cement can be partially replaced with binding additives such as ground granulated blast furnace slag or silica fly ash. Adding a binding additive to the concrete mix has the potential to reduce CO_2_ emissions and the cost of concrete production. As reported by Jones et al., replacing cement with 20, 35, and 55% fly ash can lead to a carbon dioxide reduction of 19.4%, 45.8%, and 53.7%, respectively [1,2,3,4].

The carbon dioxide emission limits introduced by the European Union (target: 55% emission reduction by 2030) encourage research into next-generation materials containing less clinker. Currently, siliceous fly ash from hard coal combustion is widely used in cement technology, mainly in concrete technology. The wide application siliceous fly ash is mainly determined by its high fineness, similar to cement, their chemical and phase composition, and their pozzolanic activity. The use of silica fly ash to produce concrete is only possible when the requirements specified in PN-EN 450-1:2012 are met [5,6,7].

The development of sewage networks and thus the municipal sewage treatment plants observed in recent years causes the formation of an increasing amount of sewage sludge. Municipal wastewater supplied to wastewater treatment plants is primarily a mixture of domestic and industrial wastewater, which is also supplied with rainwater and water infiltrating from the ground. The qualitative and quantitative characteristics of such wastewater depend on the type and condition of the sewage system, the amount of water used, the industrialization of the city, or the standard of living of the inhabitants. Both the quantity and composition of the wastewater flowing into wastewater treatment plants generally change in the daily, weekly, monthly, and yearly cycles [8]. Considering the ban on the storage of sludge since 1 January 2016, their management has become not only a technical and economic problem, but also an ecological problem [9]. Until now, the generated sewage sludge was landfilled or, after initial stabilization (aerobic, anaerobic, with lime), was released into the environment, e.g., as fertilizers or for earthworks. However, the sanitary danger, high weight, and hydration of generated sewage sludge, have always been a technical problem. Considering that the presence of toxic substances and heavy metals in generated sewage sludge limits the possibility of its use in agriculture, thermal methods are the most appropriate methods of sewage sludge disposal [10,11,12,13,14]. As a result of this process, the volume of waste (sludge) is reduced, the heat or electricity is obtained, and the content of sulfur and nitrogen compounds in the exhaust gas is reduced. The secondary waste material generated in sewage sludge thermal treatment installations is waste (fly ash—Fly ash sewage sludge—SSA) with the code 19 01 14, which also requires appropriate management. According to the idea of circular economy, a near-zero emission economy, ash from sewage sludge should be treated as a potential product [15].

The indication of appropriate waste management methods after the thermal treatment of sewage sludge is of particular importance in the building materials industry. There are five main trends in the literature on the use of such fly ash. They mainly concern the use of fly ash from sewage sludge as an active additive to inorganic cement binders—concrete and mortars, a component of light sintered aggregate, and a component of excess raw materials to produce construction ceramics as well as a component of a raw material mixture to produce cement or a substitute for cement/sand in earth road structures [16,17,18,19,20,21]. It should be noted, however, that their application is associated with limitations resulting from the provisions in force in Poland (Journal of Laws 2016, item 108), which in terms of their regulation, implement the Directive of the European Parliament and of the Council [EU/2010/75], which enforce rules regarding ash with a different (from fly ashes from hard coal and brown coal combustion) physico-chemical composition and, most importantly, with the fact that such ash also contains pollutants—heavy metals [22,23].

The research on fly ash from the thermal treatment of sewage sludge presented in the literature proves that the introduction of a certain amount of SSA into the concrete mix does not adversely affect the strength parameters and frost resistance of mortars and concretes. Fly ash is treated as a partial binder replacement or as a mineral additive [24,25,26,27]. In studies conducted by Monzo et al. [28], it was shown that mortars containing up to 30% SSA and that were cured in water at a temperature of 40 °C do not show a reduction in compressive strength compared to with the addition of ash. The changes the in strength of mortars are related to the pozzolanic properties of the SSA, maturation conditions, and the content of C3A. Ing et al. [29] found that cement mortars in which cement was replaced with ash in an amount of up to 10% were characterized by a similar or higher compressive strength than conventional mortars. In the studies of Cyr et al. [30], it was estimated that the fly ash introduced into the mortars in the amount of 25% and 50% caused a decrease in the compressive and bending strength parameters compared to the reference mortars. Lin et al. [31] analyzed the influence of fly ash on the compressive strength of building materials. They found that the additive with a finer graining is characterized by a higher index of pozzolanic activity, which resulted in obtaining higher compressive strength from the tested composites. In the studies of Baeza-Brotons et al. [32], ash was added to the cement composite in the amounts of 5, 10, 15 and 20%. It was found that ash influences the absorption of water contained in the composite. The pozzolanic properties of fly ash from the tarmac treatment of sewage sludge and their chemical composition are similar to traditional mineral additives. Fly ash from sewage sludge, considering the fulfillment of both environmental and technical criteria, as stated by other authors [25,26,33,34,35,36] in the amount of 5–20%, can be successfully used as an active additive for cement production. In concrete, they show an analogy to traditional mineral additives.

Test methods using modal analysis have been used for many years in technical mechanics. In construction, this methodology is less widespread. In the works of many authors [37,38], the possibilities of using this methodology in the evaluation of research on mechanical properties and its composition in various structures and elements of technical mechanics have been described. Later authors such as [39,40] organized research to adapt this research methodology to civil engineering. Many years of laboratory tests and tests conducted on real building structures have resulted in the creation of a methodology dedicated to the use of materials and building elements, including concrete elements, in testing.

The research shown in first part of the article shows the possibility of reusing fly ash from the thermal conversion of sewage sludge as an additive to concrete and to analyze the impact of the physico-chemical properties of this ash on the compressive strength of concretes produced with their participation, in accordance with the assumptions of the circular economy. The obtained test results were compared to the blank sample, which did not contain any fly ash. The main goal in this article was to conduct experimental laboratory research with the use of the modal analysis vibration methodology to determine the possibility of a non-invasive concrete mix composition by distinguishing the percentage content of plastic ash in the mix.

## 2. Materials and Methods

For laboratory tests, a concrete mix of C20/25 class with a K2 consistency was designed according to PN-EN 206 + A1: 2016-12 using the computational and experimental method according to Bukowski [41,42]. The mixture was prepared using CEM I 42.5R Portland cement (Cement Ożarów S.A., Ożarów, Poland), 0–2 mm fine aggregate (Warsaw, Poland) and 2–16 mm coarse aggregate (KIM Sp.z o.o. Warsaw, Poland), water, and an additive—fly ash from thermal treatment of sewage sludge at the “Płaszów” sewage treatment plant in Krakow (Poland). In each composition of the concrete mix, the same composition of the fine aggregate selected by the sieve analysis method and the same composition of the course (gravel) selected by the iteration method (Table 1) were adopted.

To determine the effect of fly ash on the selected mechanical properties of ordinary concretes, two types of samples were prepared:

CO—no additive;

FA—with the addition of fly ash from thermal treatment of sewage sludge in the amounts of 5% to 20%.

The physical and chemical properties as well as the phase composition of CEM I 42.5R Portland cement in accordance with the requirements of PN-EN 197-1:2012 [43] are presented in Table 2 and Table 3.

The composition of the prepared concrete townspeople per 1 m^3^ is presented in Table 4.

To assess the effect of fly ash from the thermal transformation of sewage sludge on the properties of concrete mix and concrete, the following were tested: apparent density (PN-EN 12350-6: 2011), air content (determined by the pressure method (PN-EN 12350-7: 2011)), and consistency (determined by the method fall cone (PN-EN 12350-2: 2011)). Compressive strength was tested in a hydraulic testing machine H011 Matest (Italy) in accordance with the guidelines contained in PN-EN 12390-3: 2019-07 after 28, 56, and 356 days of maturation [44,45,46,47,48]. For each series of tests, 15 samples with the dimensions 10 × 10 × 10 cm were prepared.

**Figure 1:**
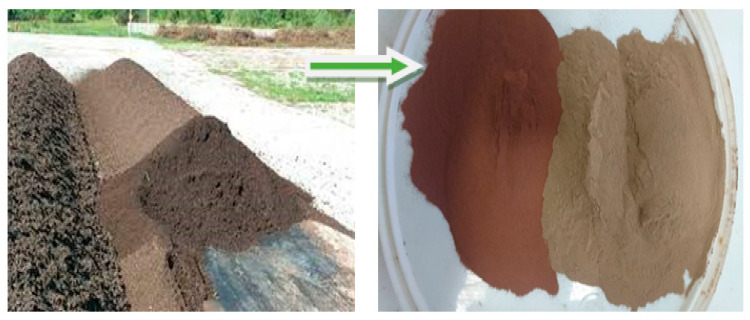
Converted sludge to fly ash from the thermal treatment of sewage sludge—SSA.

The municipal wastewater that is delivered to the treatment plant is a mixture of industrial and domestic wastewater, which is additionally supplied with rainwater and infiltrating water from the ground. The amount and composition of the wastewater flowing into the wastewater treatment plant is subject to changes during daily, weekly, monthly, and yearly cycles. Additionally, their qualitative and quantitative characteristics primarily depend on the condition and type of a given sewage system, the standard of living of the inhabitants, the amount of water used, or the industrialization of the city. An important rule is that there is no typical composition and quality of municipal wastewater; thus, there is no typical composition of sewage sludge—fly ash generated during thermal transformation—Figure 1. To determine the physico-chemical properties, tests were conducted on fly ash from the thermal transformation sewage sludge.

Pozzolanic activity determination was conducted in accordance with PN-EN 450-1: 2012 and ASTM C379-65T. The chemical composition of the material was determined by the X-ray energy dispersion fluorescence (XRF) method on an Epsilon 3 spectrometer (Panalytical, Eindhoven, The Netherlands). The study was conducted in the measuring range of Na-Am using an apparatus equipped with an X-ray Rh 9W lamp, working at 50 kV and 1 mA, a 4096-channel spectrum analyzer (Panalytical, Eindhoven, The Netherlands), six measurement filters (Panalytical, Eindhoven, The Netherlands) (Cu-500, Cu-300, Ti, Al-50, Al-200, Ag) and a high-resolution semiconductor SDD detector (Panalytical, Eindhoven, The Netherlands) (Be window, 50 µm thick) cooled with a Peltier cell. Particle size distribution analysis was performed based on the laser diffraction phenomenon using the Mastersizer 3000 analyzer (Malvern Panalytical, Malvern, UK). The measurement was conducted in a dispersing liquid (demineralized water) in the presence of an ultrasonic probe in order to break up the larger aggregates from the tested samples. Grains with equivalent diameters ranging from 0.1 µm to 1000 µm were analyzed. Morphology and chemical composition in the micro-area determination was conducted using the SEM Quanta 250 FEG scanning microscope (Panalytical, Eindhoven, Netherlands) by FEI, which was equipped with the chemical composition analysis system based on the energy dispersion of X-rays—EDS (Energy Dispersive X-Ray Spectroscopy), by EDAX. Mineral composition was determined using X-ray phase analysis (XRD). Measurements were made using the powder method using a Panalytical X’pertPRO MPD X-ray diffractometer with a PW 3020 goniometer (Panalytical, Eindhoven, Netherlands. A Cu copper lamp (CuKa = 1.54178 Å) was used as the X-ray emission source. X’Pert Highscore software (Panalytical, Eindhoven, Netherlands) was used for processing the diffraction data. The identification of the mineral phases was based on the PDF-2 release 2010 database formalized by JCPDS-ICDD [7,27,49].

**Figure 2:**
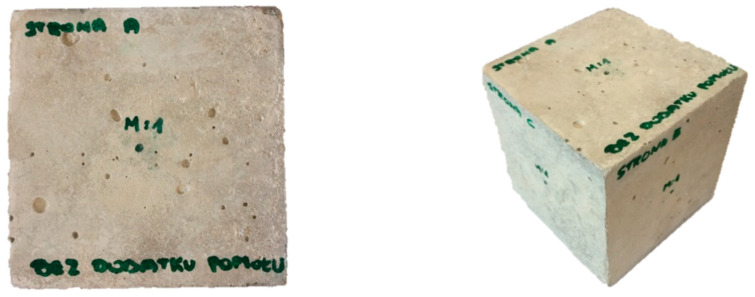
Ordinary concrete cube.

**Figure 3:**
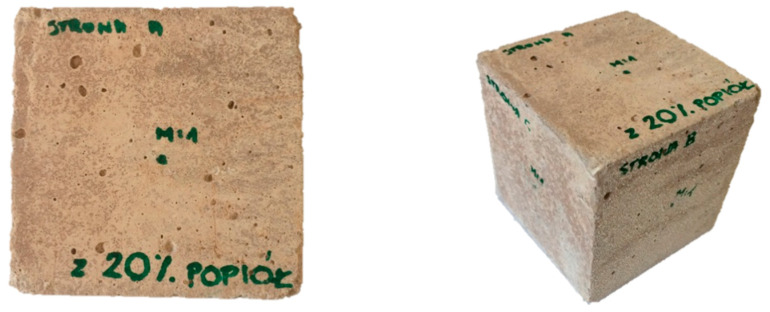
Ordinary concrete cube with the addition of 20% fly ash from sewage sludge.

After conducting the strength tests for the purpose of vibration tests using the modal analysis methodology, standard samples were prepared. In the first case, the cube was made from an ordinary concrete cube, while in the second case, the material composition was modified by adding 20% fly ash from sewage sludge ash. A characteristic feature of this change is the color of both samples, which is shown in Figure 2 and Figure 3. In both cases, the tested cube had the same external dimensions (10 cm edge) and weight (approx. 2.47 kg). This was the first time that a pilot study was conducted using the modal analysis methodology and aimed to confirm the hypothesis of whether vibration methods can be used in the assessment of the amount of the admixture used in concrete and the effect that it has on the properties of concrete.

### 2.1. Types of Modal Analysis

The modal analysis of mechanical systems is a method of examining dynamic properties consisting of tracking changes in the parameters of the modal structure or wall element model under test resulting from wear, damage, or failure and is based on current observations of the object. In this method, a modal model is created in the form of the natural frequencies set, the vibration modes, and the damping coefficients for an object without damage as a pattern. Then, during operation, the modal model is identified, and its correlation with the model for an undamaged object is examined. When such a correlation occurs, it can be stated that the object is fit. In the absence of correlation, the object is in a state of unfitness, caused, for example, by aging or damage [38,39,50,51,52].

Based on the knowledge of the modal model, it is also possible to predict the response of an object to any disturbance, both in the time and frequency domains. It is implemented as a theoretical, experimental, or operational modal analysis.

Theoretical modal analysis is defined as the intrinsic problem of a matrix, which is dependent on the matrix of masses, stiffness, and damping. Theoretical modal analysis requires solving the problem for the adopted structural model of the structure under study [37,40,53]. The determined sets of eigenfrequencies, damping coefficients for the eigenfrequencies, and the modes of free vibrations allow for the simulation of structure behavior with any input, the selection of the controls, structure modification, among other features. It is used in the design process when it is not possible to conduct research on a similar object.

It is especially useful in the analysis of the resistance of structures to various disturbances and enables the assessment of skyscrapers, bridges, railway wagons, vibrations, and tectonic movements. An example of the above may be a model of a skyscraper structure—a system of heavy plates connected to each other by relatively flexible girders. So far, in practical application, modal analysis has been used to diagnose lattice structures (masts, antennas, cranes), diagnose a turbine set, and diagnose the quality of bridge structures. In most of these applications, it is assumed that because of failure, the stiffness of the structure changes locally, which causes changes in the parameters of the modal model. By tracking changes in the mode of natural vibrations, it is possible to determine the area in which significant destruction occurs [54].

The sets of the eigenfrequencies, the damping coefficients for the eigenfrequencies, and the modes of free vibrations determined in the eigenvalues problem allow for the simulation of the behavior of the structure under any excitation, control selection, structure modification, among other factors.

The analysis of the eigenfrequencies and eigenvectors is obtained from the equation of motion (after omitting the terms containing the damping matrix and the vector of external loads). Then, the equation of the natural vibration motion has the following form:

(1) $$B\ddot{q}+Kq=0$$

For a system with one degree of freedom, the solution has the form of a function:

(2) $$q\left(t\right)=\vec{q}sin\left(\omega t+\varphi \right)$$

where *q* is the vector of the natural vibration amplitudes.

Substituting the above equation and the second derivative into the equation of motion, we achieve

(3) $$\left(-\varpi^{2}B+K\right)\vec{q}\sin\left(\varpi t+\varphi \right)=0$$

The above equation must be fulfilled for any moment t—then, the system of algebraic equations is obtained:

(4) $$\left(K-\varpi^{2}B\right)\vec{q}=0\;\left(k_{11}-\omega^{2}m_{11}\right)q_{1}+\left(k_{12}-\omega^{2}m_{12}\right)q_{2}+\ldots +\left(k_{1n}-\omega^{2}m_{1n}\right)q_{n}=0\;\left(k_{21}-\omega^{2}m_{21}\right)q_{1}+\left(k_{22}-\omega^{2}m_{22}\right)q_{2}+\ldots +\left(k_{2n}-\omega^{2}m_{2n}\right)q_{n}=0\;\left(k_{n1}-\omega^{2}m_{n1}\right)q_{1}+\left(k_{n2}-\omega^{2}m_{n2}\right)q_{2}+\ldots +\left(k_{nn}-\omega^{2}m_{nn}\right)q_{n}=0$$

The result is a linear system of homogeneous algebraic equations that has a non-zero solution only when

(5) $$\det\left(K-\varpi^{2}B\right)=0$$

After transformations, we obtain an nth degree polynomial with respect to $\omega^{2}$. Multiple roots can occur among the roots, and the vector made up of frequencies set arranged in the increasing values order is called the frequency vector, and the first frequency is called the fundamental frequency [39].

(6) $$\omega =\left[\omega_{1},\omega_{2}\ldots ,\omega_{n}\right]$$

Theoretical modal analysis is mainly used in the design process when it is not possible to conduct research on the object.

Experimental modal analysis is one of the techniques that is used for identifying structure modal parameters. Experimental modal analysis is a technique that is often used in practice for examining the properties of objects, both at the stage of construction and in operation. The identification experiment in experimental modal analysis consists of forcing the vibrations of the object with the simultaneous measurement of the exciting force and the response, most often in the form of a vibration acceleration spectrum. The modal model is obtained from the stabilization diagram and the animation of the vibration form presented in the software.

**Figure 4:**
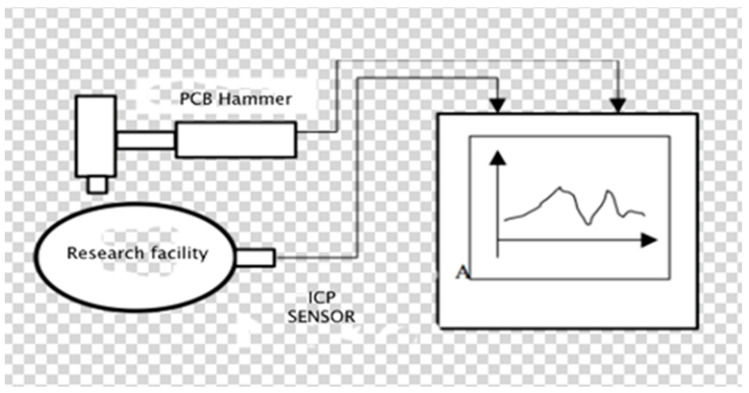
The essence of research in experimental and operational modal analysis.

The identification experiment in experimental modal analysis (Figure 4) consists of forcing the vibrations of the object with the simultaneous measurement of the exciting force and the response of the system, most often in the form of vibration acceleration amplitude.

The measurement results based on the adopted geometric model are processed in the software system (LMS, MATLAB, VIOMA), obtaining the vibration spectrum of the driving force at the input of the system, the spectrum of the vibration acceleration amplitude at the output of the system, and the stabilization diagram—from which the parameters of the modal model can be estimated [39,40]. Stabilization diagram is simply a plot of different model orders or damping ratios versus the frequencies identified at each of model orders. Generally, the typical stabilization diagram is a graph where the frequency is plotted as the X-axis and the model order as the Y -axis

**Figure 5:**
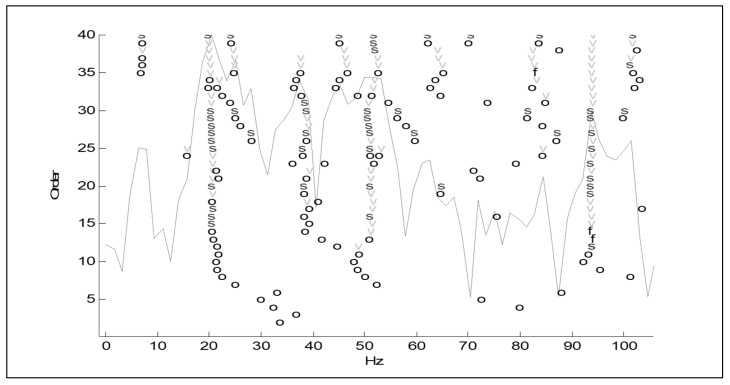
Stabilization diagrams. Notations: order—order of the pole, o—unstable pole, f—pole has a constant frequency. The v-pole has a constant frequency and modal vector; s—stable pole.

The next Figure, Figure 5, shows example stabilization diagrams with the distinguished frequencies of the free vibrations.

By subjecting the recorded vibration signal and the response of the system to the FFT (Fast Fourier Transform) analysis (fast Fourier analysis), it is possible to obtain the measure of the dynamic state FRF after the transformations. The function (FRF- Frequency Response Function) is a basic measure that describes the global dynamic properties of the structure under study. The FRF describes an input–output relationship between two points on a structure (material) as a function of frequency.

The experimental parameters of the modal model (natural frequency, damping, and vibration modes) as well as many other important vibration estimators can additionally be obtained without major difficulty in the procedures accompanying the determination of the FRF.

Operational modal analysis is used to identify objects of large spatial dimensions and large masses. It is based on the measurement of the response to operational forces, resulting from external forces or kinematic excitations and the process of destruction of the building elements [40].

Operational modal analysis:

- Enables the analysis of large-sized objects for which laboratory tests are difficult;
- Models objects more correctly because the inputs correspond to real loads;
- Enables the identification of nonlinear models.

**Figure 6:**
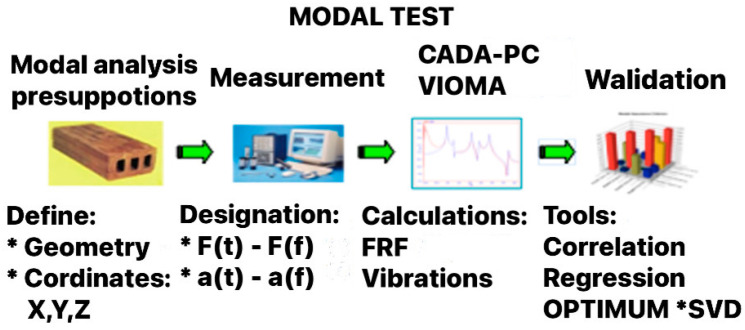
The essence and scope of research in operational modal analysis.

For determining the vibration form, the operational analysis method is based on a multi-channel response measurement at the modal points of a real object. The system enables a graphic representation of the object’s behavior under operating conditions. The input data to the system are the time courses of mechanical vibrations occurring at the modal points of the object that are related to one of them (with the highest amplitude). The essence of applying operational modal analysis is shown in Figure 6.

Operational modal analysis is a technique based on the measurement of the system’s response to unknown operational forces resulting from the action of technological process forces or kinematic excitations and the process of structural element destruction. During the tests, the measurement points and reference points should be determined and should be constant during the measurements. In this method, based on the measured signals at the facility exit obtained during the measurements at the selected reference and measurement points for unknown system inputs, the estimation of the modal parameters is performed. As a result of the estimation, the poles of the system are identified, and then, on their basis, free vibration modes are estimated. In this method, the selection of the number and the location of the reference points becomes important.

### 2.2. Measurement Systems

**Figure 7:**
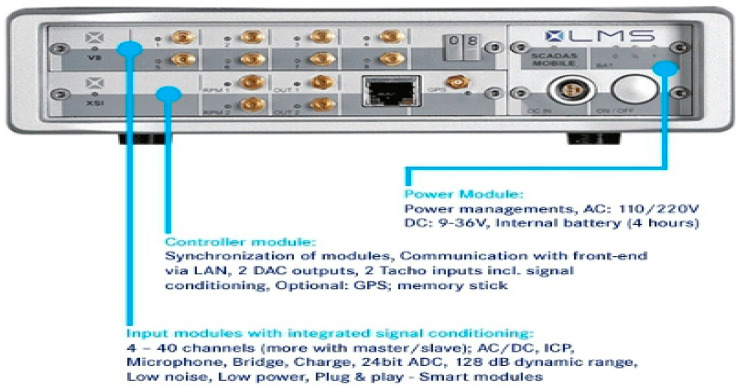
LMS SCADAS Recorder.

The LMS SCADAS Recorder system (SIEMENS LMS TEST EXPRESS, Leuven, Belgium) is one of the most advanced measurement systems used in research on object condition degradation. A LMS SCADAS Recorder is a device that combines the features of an analyzer and a classic recorder. Figure 5 shows the front panel of the LMS SCADAS system. Depending on the specifics of the tests, the user configures the device with the necessary set of measurement cards with the appropriate number of measurement channels. Contrary to classic LMS recorders, a SCADAS Recorder is fully automatic and does not need to be computer or remote controlled to conduct the process of recording the measurement signals, and the data are saved on a Compact Flash card. This device is fully compatible with professional engineering software, and it is shown in picture below (Figure 7).

LMS Test.Lab is a system for measurement, data acquisition, analysis, and reporting. The system includes dedicated procedures for structural and acoustic testing, environmental testing, vibration control, and quality testing.

LMS Test.Lab is used to initially provide data collected on real objects and to integrate them into the simulation process. It can be used to provide simulation software with data on dependent models that are either too difficult to implement or that would take too long to create. These data can be, for example, time waveforms, spectral FRF transition functions, cross power waveforms, etc. After testing the prototype created based on the simulation, we can test it, and LMS Test.Lab will provide us with data for its modification and improvement. The only way to obtain the best results is a combination of simulations and tests on real objects that were based on them [37,40].

Test.Lab is a multi-functional software built modularly so that the program can be adapted to specific measurements. The software configured during the modal analysis allows you to create geometry, calibrate sensors, and determine appropriate measurement ranges; control inductors; record the inputs, responses, and estimates; select modal parameters; and visualize modal forms.

The LMS Test.Lab measurement system is complemented by the LMS Virtual.Lab system, which allows you to combine the most important aspects of simulation and tests on existing facilities. It offers a unique, hybrid approach to simulation—input data from real objects are combined with data from simulated objects.

Presented issues indicate that the use of technical diagnostics is important at all stages of the existence of facilities and is particularly useful at the stage of their operation, providing information and data for the study of the constructed models of operational strategies.

The condition of actual building structures or masonry elements should be tested in a simple and effective method, using the minimum number of measurements. In laboratory tests of building elements, experimental modal analysis is used for this purpose, and in tests of entire structures, operational modal analysis is used. In both modal analysis cases, the most modern measuring equipment, purchased for research projects conducted by means of LMS—under the name LMS TEST.XPRESS—was used to measure the time courses of the excitation and the system response to the given excitation and to determine the main measures of quality (FRF and Coherence functions). This software allows you to easily conduct, according to the developed degradation state analysis algorithm, modal analysis of masonry elements and any other building structures.

The LMS Test. Xpress 4A software (SIEMENS LMS TEST EXPRESS, Leuven, Belgium) allows for the registration of the input force signal and the response in the form of time waveforms and can generate a cross-correlation function, which, in turn, can generate a stabilization diagram with natural vibration frequencies for each structure element.

Correct measurement depends on obtaining the level of the exciting force that was previously determined and on the appropriate level of the response signal. Repeatability tests of waveforms concerning measures of general changes in the state of material destruction, the construction of stabilization diagrams, and the determination of the values of the areas under the FRF and coherence curves are just some of the advantages of the LMS program.

## 3. Results and Discussion

### 3.1. Properties of the Fly Ash

Fly ash from coal combustion is currently an important and valuable raw material used in the production of cement or concrete. The applicable standards define the requirements for the physical properties and chemical composition of ash that can be used as an additive to cement or concrete [7]. An important ash parameter that determines its use in concrete technology is its phase and chemical composition, pozzolanic activity index, and fineness. According to the requirements of the standard, the total content of silicon dioxide, iron oxide, and aluminum should be a min. of 65% by weight, with a reactive SiO_2_ content of at least 25% by weight. Additionally, the content of reactive CaO should not exceed 10% MgO 4%, and the total alkali content, calculated as Na2O content (equivalent), should not exceed 5% by weight. The content of soluble phosphate (P2O5) should not exceed 100 mg/kg. The activity index after 28 days of maturation should reach the value of ≥75%, and after 90 days, the value of ≥85% should be reached. The pozzolanic activity of fly ash from the thermal treatment of sewage sludge after 28 days of maturation was 66.4%, while after 90 days, it was 77.3%. Additionally, it should be emphasized that the PN-EN 450-1: 2012 standard refers to ash from hard coal combustion—silica fly ash [7].

**Figure 8:**
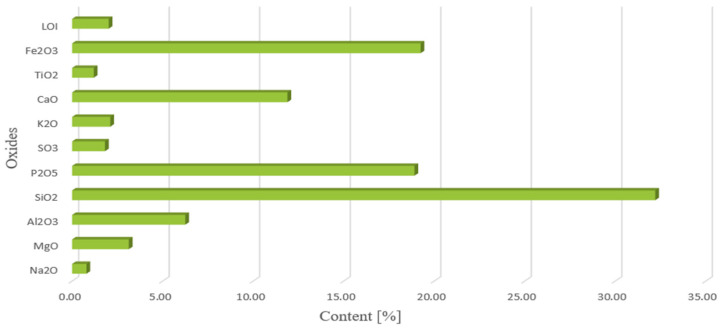
Oxide composition of fly ash from thermal treatment of sewage sludge.

The results of the analysis of the oxide composition of fly ash from the thermal transformation of sewage sludge are presented in Figure 8.

It was observed that the sum of the content of aluminum oxide (Al_2_O_3_), silicon dioxide (SiO_2_), and iron oxide (Fe_2_O_3_) in the fly ash from sewage sludge was at the level of 57.71%. The largest percentage of the ash was silicon dioxide (32.21%), iron oxide (19.25%), phosphorus (18.91%), and calcium (18.64%). The loss on ignition, which expresses the content of unburned carbon in the tested sample of fly ash in the fluidized bed furnace at a temperature above 850 °C, was only 0.5%. The phosphate content was also much higher than that of conventional ash. This is due to the removal of phosphorus from the wastewater and its accumulation in the sludge.

**Figure 9:**
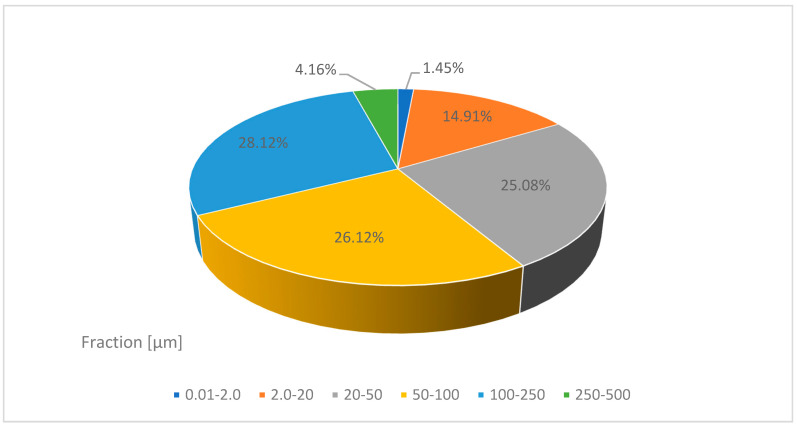
Volumetric distribution of the particle fractions of fly ash from thermal treatment of sewage sludge.

Figure 9 shows the volume distribution of the individual particle fractions of the additive used. At the largest percentage share above 25%, there were grains with a diameter of 20–250 µm, while at the smallest percentage share, 1.45%, there were grains with a diameter of 0.01–2.0 µm. For comparison, in the research conducted by Kosior-Kazeruk, the content of particles (0.25 mm) in ash from the thermal treatment of sewage sludge was 7% [55]. According to Joshi and Loftia, the diameter of the ash grains ranges from 1 to 150 µm and is similar to the grains of the binder—cement [56].

**Figure 10:**
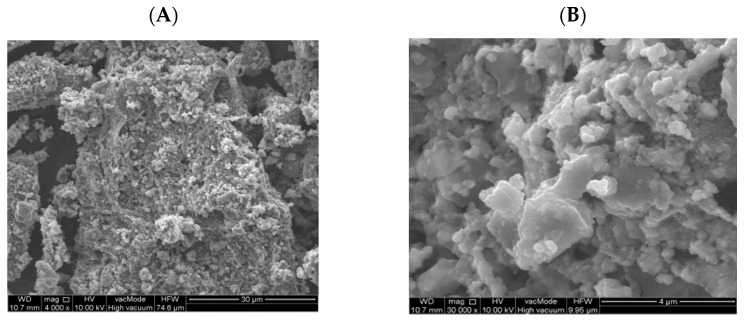
SEM photo of fly ash from thermal treatment of sewage sludge in enlargement. (**A**)—4000 times, (**B**)—30,000 times.

**Figure 11:**
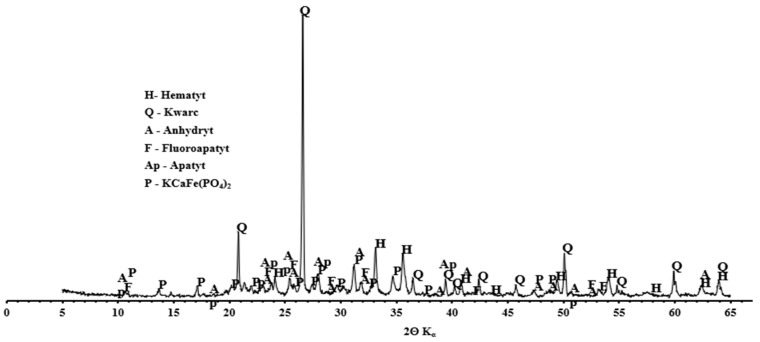
Mineral composition of fly ash from the thermal treatment of sewage sludge.

Figure 10 shows a SEM photo of fly ash from the thermal treatment of sewage sludge. Chemical analysis in the SEM-EDS micro-area showed the presence of grains with the chemical composition of calcium, aluminum, iron, and phosphorus as well as potassium, magnesium, and sodium. However, considering the mineral composition of ash from sewage sludge, hematite, quartz, and anhydrite were dominant (Figure 11). The fineness of the ash from sewage sludge determined according to PN-EN 451-2: 2017-06 was 50.7%, while the specific density tested according to PN-EN 1097-07: 2008 was 2534 kg/m^3^ [57,58].

Fly ash samples are predominantly irregular grains with variable size and a strongly developed surface, showing a loose and rough structure of the material that also has high porosity. It leads to higher water absorption, which is connected to the increased water demand of concrete containing SSA. This finding is consistent to that of Alonso and Wesche [59], who wrote that ash grains with a diameter of over 125 µm are highly porous and that the Blaine number (SSA) of these materials falls into the range 250–550 m^2^/kg. As Malhotra and Ramezanianpour demonstrated, the grain size and SSA of fly ash is not associated with the formation source. Spherical and cuboidal forms are very rare. Grains of fly ash produced in coal combustion are usually spherical, but they can be irregular or angular [60].

### 3.2. Properties of the Concrete Mix

The basic properties of each of the prepared concrete samples were checked: consistency, density, and air content. Based on the results, it was found that the fly ash from the thermal treatment of sewage sludge affects its individual parameters. The lowest air content was observed for a sample of the reference concrete mixture CO, with the air content being equal to 1.8%, while the highest air content occurred in the mixture in which 20% of the cement was exchanged with ash, FA20%, which had an air content equal to 2.9%. The density of the concrete mix ranged from 2322 to 2394 kg/m^3^. Concrete mix without additive obtained a plastic consistency, similar to concrete mixes containing different amounts of additive.

### 3.3. Compressive Strength

**Figure 12:**
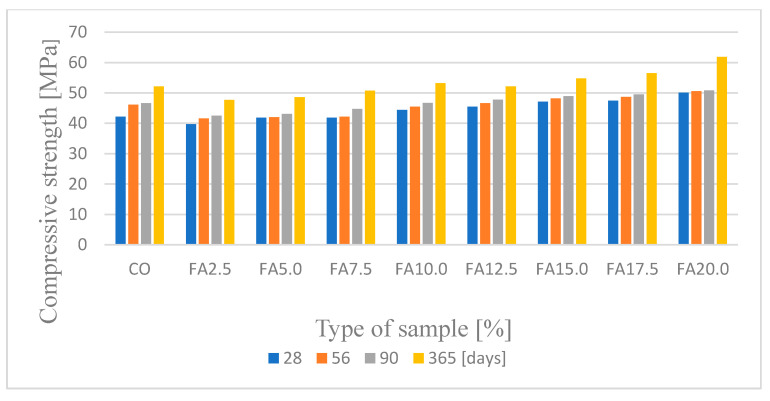
Average compressive strength in different periods of concrete maturation.

The results of the compressive strength test in the four maturation periods (28, 56, 90, and 365 days) of concrete samples with different fly ash content from the thermal treatment of sewage sludge are shown in Figure 12.

Fly ash from the thermal treatment of sludge does not meet the requirements of PN-EN 450-1: 2012, but replacing cement with this additive in the amount of up to 20% had a positive effect on the compressive strength. The highest compressive strength in the first maturation period (28 days), equal to 50.1 MPa, was achieved by the FA20% concrete samples, while the lowest strength, equal to 39.7 MPa, was achieved by the samples in which the cement was replaced with 2.5% ash. Compared to the reference concrete, the decrease in concrete FA5% was 5.6%, while the increase in concrete strength FA 20% was 18.7%. Taking into account the subsequent maturing periods, i.e., after 56, 90, and 365 days, a further increase in the compressive strength of all of the samples was observed. After 28 courses, the compressive strength FA20% was the highest in all of the studied maturation periods. These values were 50.6 MPa, 50.8 MPa, and 61.9 MPa. The increase in endurance between days 28 and 365 of puberty was 23.6%. The lowest compressive strength was observed for the FA2.5% concrete samples. After 56 days, the strength of these samples was 41.6 MPa and 42.5 MPa after 56 days and 47.7 MPa after 365 days. The increase in strength between the first and last tested maturation period for these samples was 12.6%. In addition, it was observed that the reference concrete showed a lower compressive strength than the FA20% samples by a value equal to 18.8% after one year of maturation. Our own experimental research conducted on the influence of fly ash from the thermal transformation of sewage sludge on the compressive strength in different maturation periods confirms the positive influence of this type of ash [25,26,27].

According to Williams [61], a low loss of ignition and the P2O5 content affect the strength of concrete. In addition, it is assumed that the presence of phosphate ions may cause a slow increase in the strength of concretes containing ash due to the delay in the cement hydration process [62]. The diversified chemical composition of different types of fly ash (Figure 8) allows for the production of ash concretes that meet the standard strength requirements. The pozzolanic and hydraulic properties as well as the chemical composition (silica, iron, aluminum, calcium, magnesium, phosphorus, and oxygen) of sewage sludge ash used in concrete as a replacement for Portland cement are analogous to traditional mineral additives [63]. Chang et al. [64] showed that the addition of ash from the thermal treatment of sewage sludge increases the water absorption capacity and reduces the workability and compressive strength. The most favorable conditions were obtained with the incorporation of 10% ash into the mixture. In our own research, similar results were obtained. Thus, the compressive strength of concrete with the 10% addition of sewage sludge ash was higher than for the samples with 5% and 15% ash content. It should be added here that the compressive strength of this sample was only lower than that of concrete with a 15% share of silica ash [26]. The optimal ash content from sediment, according to information provided by other authors, in cement materials ranges from 5% to 20% [34,35]. Taking environmental and technical criteria into account, Chen et al. [65] analyzed the possibilities of using ash as a substitute for cement and/or a sand substitute in building materials. It was confirmed that mortar containing 10% ash has a compressive strength that is similar to that of a conventional mortar. Replacing cement with ash in the amount of 25% delays the process of setting the slurry and slows the increase in the compressive strength of mortars and concretes compared to reference composites without the addition of ash. However, by extending the maturation time of the composites, it is possible to obtain the strength required for construction concretes [55,66]. An important and binding rule is that there is no typical composition and quality of municipal wastewater; thus, there is no typical composition of the fly ash that is formed during the thermal transformation of sewage sludge, which hinders the common use of ash as active additives for concrete.

### 3.4. Modal Analysis Results of Ordinary Concretes with the Addition of Fly Ash from Sewage Sludge

**Figure 13:**
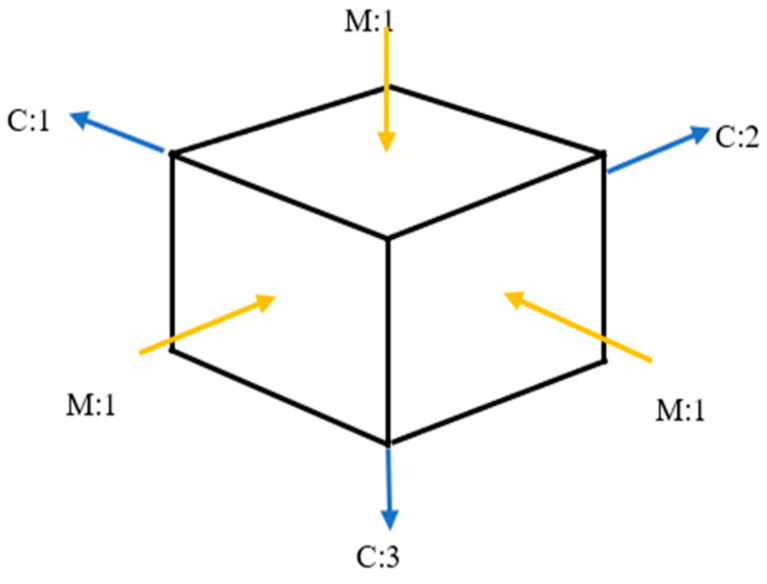
Distribution of excitations and signal response points on the research object.(C and M: the names of axels in software).

The object of the current research is a construction material in the shape of a cubic cube. In the case of the objects that were tested, the composition of the material used to make the cube changed. During the modal test experiment, four cubes were tested (two that were made of ordinary concrete and two that were concrete cubes with the 20% addition of fly ash from the sewage sludge ash addition) (Figure 13).

**Figure 14:**
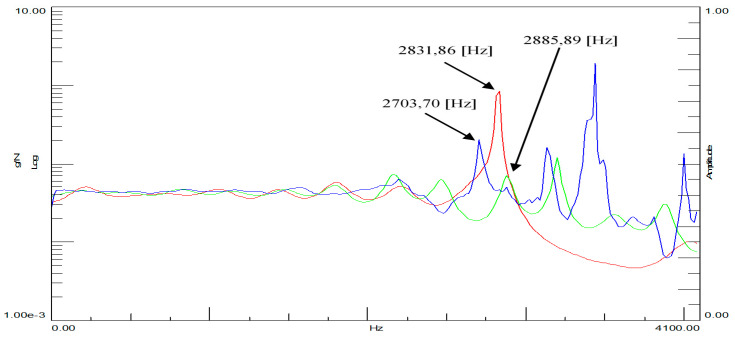
Spectral functions of the FRF transition for ordinary concrete cube. Legend: 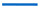 spectral transition function FRF (Side A), 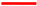 spectral transition function FRF (Side B), 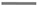 spectral transition function FRF (Page C).

**Figure 15:**
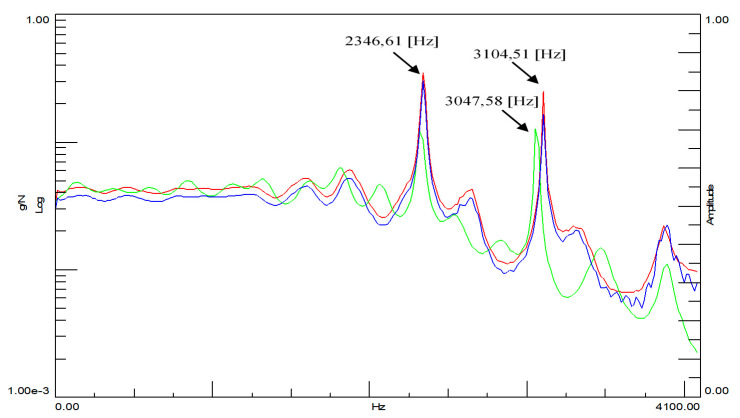
Spectral functions of the FRF transition for the ordinary concrete cube with the 20% addition of fly ash from the sewage sludge ash addition. Legend: 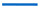 spectral transition function FRF (Side A), 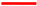 spectral transition function FRF (Side B), 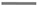 spectral transition function FRF (page C).

During the experiment, two time courses were recorded: one from the modal hammer and the other from the vibration sensor attached to the test object. In modal studies, the frequency range from 0.7 Hz to 4100 Hz was adopted. Due to the symmetry of the cube, it was decided that the modal study would be conducted in three directions, and it was because of this that three spectral transition functions were obtained for each cube. Figure 14 and Figure 15 show the FRF plots obtained from the modal study performed on both cubes.

The initial analysis of the obtained spectra focuses on the case of the cube without the addition of ash around the three natural frequencies marked in Figure 15. The situation is completely different in the case of the spectral transition functions determined for the cube with the 20% ash addition. In addition to much more convergent spectra, three characteristic vibration frequencies were also obtained. A particularly interesting situation is that in the predetermined natural frequencies, there is a very large discrepancy between the cube with and without the additive. This would initially indicate a significant differentiation of these two building materials in terms of their dynamic properties. Additionally, the analysis of individual phase shifts indicates the occurrence of the structural vibrations of at least one of the three predetermined natural frequencies for the cube without the addition of ash.

**Figure 16:**
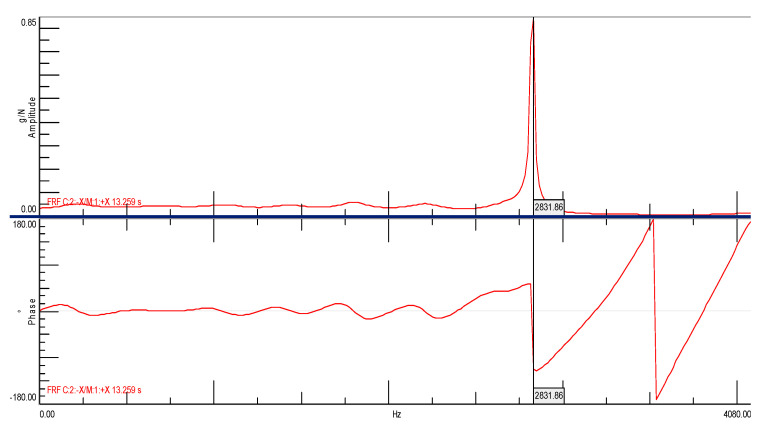
Spectral function of the FRF transition for the ordinary concrete cube with the first natural frequency marked.

**Figure 17:**
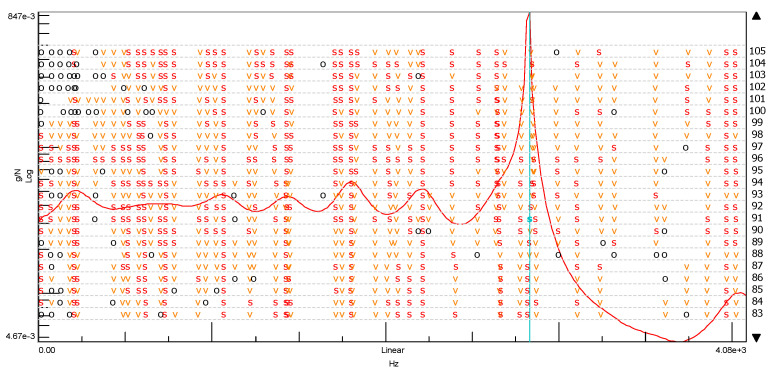
Stabilization diagram made of the spectral transition function FRF (Side B) for ordinary concrete cube (s—stable frequency field, v—modal vector, o—empty field).

Figure 16 shows the spectral transition function of the FRF in the amplitude form for a cube without the addition of ash with a marked natural frequency of 2831.86 [Hz]. It should be clearly emphasized that the determined natural frequency occurs simultaneously with the phase shift, which may be interpreted as a typical characteristic of structural vibrations (Figure 17). In Figures 16, 19 and 21 on the Spectral function of the FRF transition is shown the relevant numbers of sensors connection to the measuring apparatus (C2), the connection point of the modal hammer to inputs (M1) and the measurement directions (X and -X axes), and the time in which the measurements were performed (SUM in seconds)

**Figure 18:**
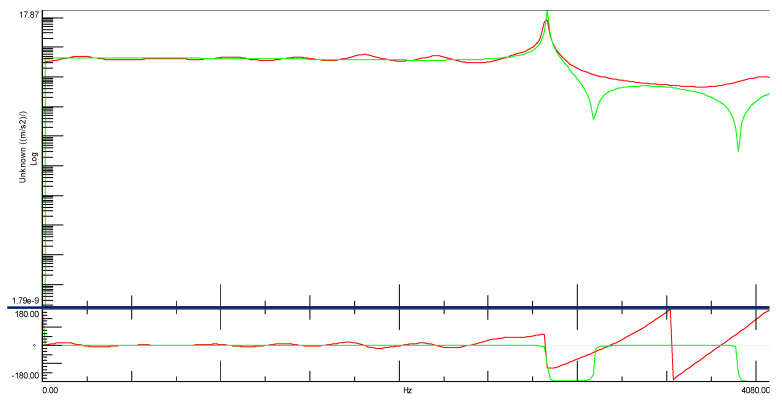
Verification of the measured spectral transition function FRF with its synthesized form.

We can easily find this frequency of vibrations in the stabilization diagram (PolyMAX estimation method—pLSCFD) created from the spectral transition function determined on the B side of the cube without the addition of ash (Figure 18).

To verify the correctness of the selected natural frequency of the cube without the addition of ash, the method of comparing the synthesized FRF spectra with the corresponding original FRF spectra was used, thus obtaining a correlation at the level of 84.77%. This indicates a very high level of correctness for the selected model. The effect of the method outside of the selected band was compensated for with the upper and lower residuals.

**Figure 19:**
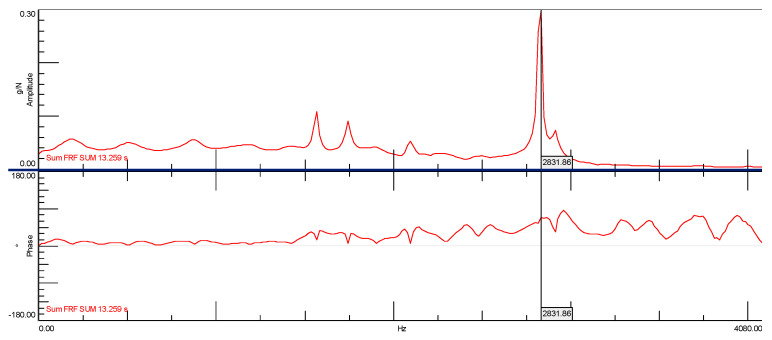
Spectral function of the FRF transition created with the use of the SUM indicator for the ordinary concrete cube.

**Figure 20:**
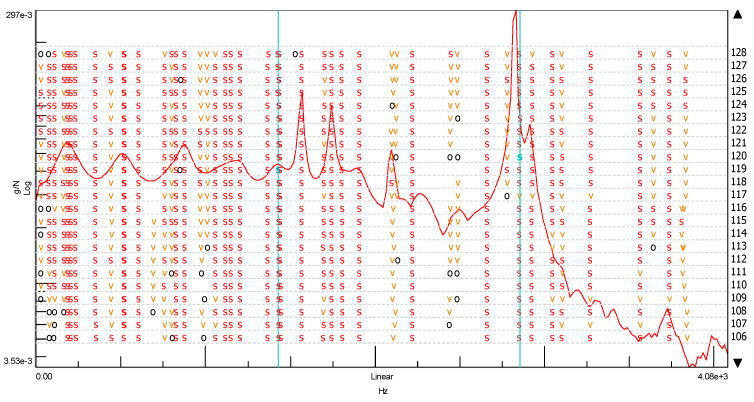
Stabilization diagram made of the spectral transition function FRF (using the SUM index) for the ordinary concrete cube (s—stable frequency field, v—modal vector, o—empty field).

In another modal study, a stabilization diagram was created considering all three spectral transition functions of the FRF corresponding to each of the three sides of the cube without the addition of ash. For this purpose, a function in the form of the SUM indicator was used, which strengthens their exposure on the chart in the places where peaks occur. This situation is shown in Figure 19 by comparing the averaged spectral function of the FRF transition with phase shifts. The graph clearly shows that the phase shifts, which are a kind of determinant in the case of selecting the appropriate natural frequencies, are not as clear as in the previous case. On this basis, a stabilization diagram was created, which is shown in Figure 20.

Additionally, in this case, the most significant natural vibration frequency is the one with the value of 2852.83 (Hz). This characteristic eigenfrequency occurs, however, in the vicinity of several other frequencies, and after multiple selection, the frequency of 1427.99 (Hz) was specified only for further analysis. Ultimately, however, due to the very high modal damping factor (exceeding 35%), it was necessary to eliminate this form from further analysis. Thus, the effect of considering all of the spectral FRF transition functions of the cube without the addition of ash in the modal analysis resulted in obtaining one characteristic natural vibration frequency. Its value, compared to the modal analysis based on one spectral transition function FRF, is shifted by approximately 21 (Hz). Therefore, it can be assumed that the first and the same time characteristic frequency of the cube’s natural vibrations without the addition of ash is at the value of 2831.86 (Hz).

**Figure 21:**
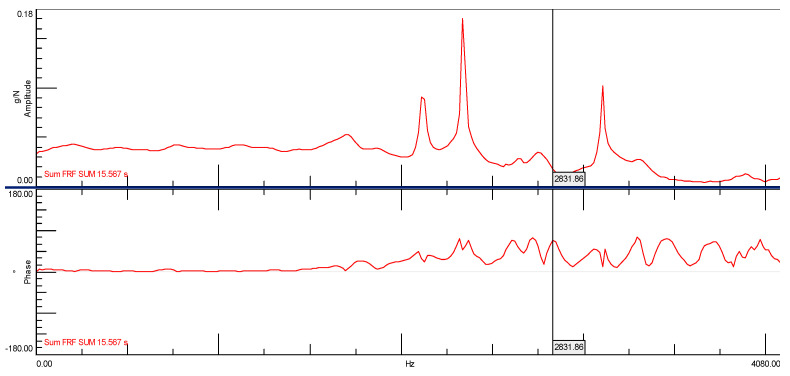
Spectral function of the FRF transition created with the use of the SUM indicator for the ordinary concrete cube with the addition of fly ash from sewage sludge.

**Figure 22:**
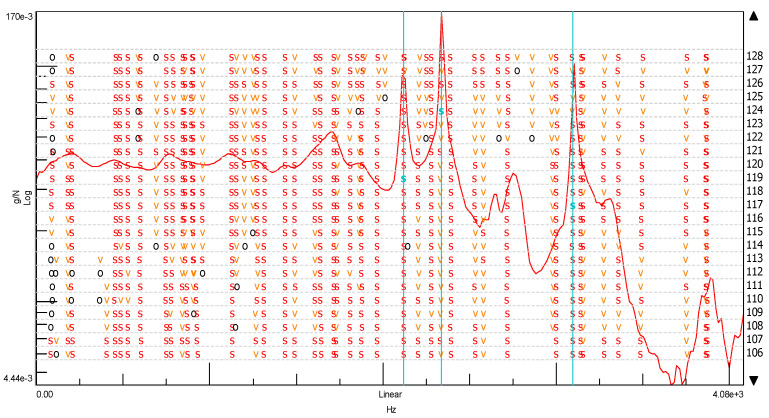
Stabilization diagram made of the spectral transition function FRF (using the SUM index) for the ordinary concrete cube with the 20% addition of fly ash from sewage sludge. (s—stable frequency field, v—modal vector, o—empty field).

Another modal analysis concerned a cube with a 20% ash addition. Considering the very high convergence of the determined spectral functions of the FRF transition for each side of the cube with the 20% ash addition, it was decided to analyze all of the FRF spectra using the SUM index. Its form is shown in Figure 21 together with the phase shifts. The diagram presented in Figure 22 shows the frequencies of the natural vibrations, which were assumed to be characteristic for the cube without the addition of ash. Thanks to this, it is clearly visible that there is a very clear difference in the characteristic frequencies of the natural vibrations of both of these objects. This difference is at the level of about 495.17 (Hz) in the direction of the beginning of the graph’s coordinate system and is 274.2 (Hz) in the direction of higher frequencies. This is a significant discrepancy beyond the tolerance associated with the measurement error.

Based on the stabilization diagram created in this way, three natural frequencies were initially determined, which describe the dynamic properties of the cube with the addition of ash. The pre-selected vibration frequencies are shown in Table 5.

**Figure 23:**
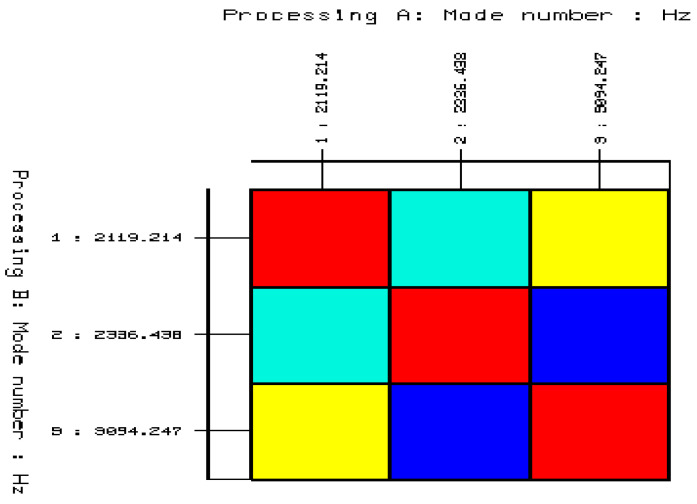
Validation of the selected stable poles and the corresponding modes of the free vibrations using the AutoMAC function (convergence percentage between the modes of on-vibrations determined from the stabilization diagrams: blue = 4% yellow = 28% red = 100% turquoise = 15%).

For selected free vibration frequencies, a validation was conducted using the AutoMAC method. The result of this validation is presented in a tabular form in Table 6 and in a graphic form in Figure 23. The validation shows that the first selected form of vibration is inappropriate for describing the dynamic properties of the cube with the addition of ash.

**Figure 24:**
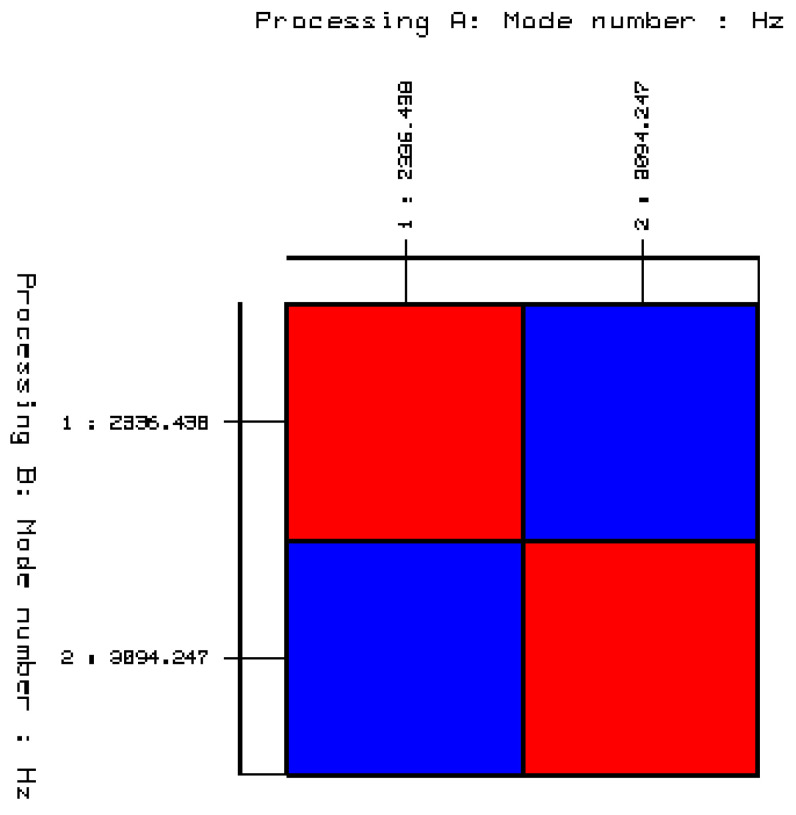
Validation of selected stable poles and the corresponding modes of the free vibrations using the AutoMAC function. (convergence percentage between the modes of on-vibrations determined from the stabilization diagrams: blue = 4%, red =100%).

As a result, the correct and final number of natural frequencies describing the dynamic properties of the cube with ash added was obtained, as shown in Table 6. Additionally, Figure 24 shows the result of the validation with the AutoMAC method.

## 4. Conclusions

The use of waste—fly ash from the thermal treatment of sewage sludge with the code 19 01 14 in building materials creates great economic benefits. The experimental studies conducted in this research showed that it is possible to use fly ash from sewage sludge to produce concrete as a partial replacement for cement. The obtained results and their analysis allow for the following conclusions:

1. There is no typical quality and typical composition of municipal wastewater supplied to the treatment plant; thus, there is no typical physico-chemical composition formed during the thermal treatment of sewage sludge—fly ash;
2. Waste-generated fly ash from the thermal transformation of sewage sludge used to produce concrete has a positive effect on its compressive strength;
3. Fly ash from the thermal transformation of sewage sludge does not meet the requirements of PN-EN 450-1: 2012. It has a different physico-chemical composition compared to the fly ash from coal combustion used in concrete technology. The highest percentage of the collected fly ash samples were oxides of silicon, calcium, phosphorus, and aluminum;
4. Concrete containing fly ash from the incineration of sewage sludge in its composition was characterized by a compressive strength comparable to that of the reference concrete without the additive. With an ash content up to 20%, they can be used as a cement substitute. The average compressive strength of concrete containing 20% fly ash from the thermal transformation of sewage sludge was after 28, 56, and 365 days of maturation was 50.1 MPa, 50.8 MPa, and 61.9 MPa, respectively;
5. The results of the conducted modal tests of cubes without and with the addition of ash showed that it is possible to distinguish both products using the method of experimental modal analysis. For the cube without the addition of ash, one natural frequency was determined at the level of 2831.86 (Hz), while for the cube with the ash addition, two natural frequencies were established at level I—2336.44 (Hz) and II—3094.25 (Hz). The obtained results of the analysis clearly indicate that the dynamic state in the case of the cube without the addition of ash and the cube with the 20% ash addition is different. This is part of an additional research study that will be continued in the future. The results of these studies are assumed to result in the development of a model for the non-invasive diagnosis of the mechanical properties of concrete containing fly ash from the incineration of sewage sludge.

## Figures and Tables

**Table 1:** Percentage of the contents of the aggregates selected by iterations.

Fraction	Fraction Mixing Percentage Ratio (for Sand and Gravel)			Grain Composition of (%)	
	I Stage	II Stage	III Stage	Sand	Gravel
0.0–0.125			30	1.26	0.38
0.0125–0.25				12.33	3.70
0.25–0.50				36.35	10.91
0.50–1.0				33.12	9.94
1.0–2.0				16.94	5.07
2.0–4.0		35	70		24.50
4.0–8.0	49	65			22.29
8.0–16.0	51				23.21

**Table 2:** Physical properties and phase composition of cement CEM I 42.5R.

Beginning of Binding Time (min)	Compressive Strength after 2 Days (MPa)	Compressive Strength after 28 Days (MPa)	Blaine’s Specific Surface Area (cm^2^/g)
218	22	49.8	3330
The share of mineral phases CEM I (5 mass)			
C_3_S—61.8	C_2_S—12.3	C_3_A—7.6	C_4_AF—4.1

**Table 3:** Chemical properties of cement CEM I 42.5R.

SiO_2_ (%)	Sulfate Content SO_3_ (%)	Roasting Loss (%)	Chloride Content Cl (%)	Alkali Content Na_2_O_eq_ (%)
20.20	3.20	3.20	0.06	0.73
CaO	Al_2_O_3_	Fe_2_O_3_	MgO	CaO_w_
65.21	4.34	2.37	1.51	1.76

**Table 4:** Concrete mix proportions by weight.

Specification	Mass of Concrete Ingredients (kg/m^3^)			
	Aggregate	Water	Cement	Fly Ash
Concrete CO	1812.15	193.55	380.12	-
FA 2.5% Concrete with quantity 5% of fly ash	1812.15	193.55	370.62	9.50
FA 5% Concrete with quantity 5% of fly ash	1812.15	193.55	361.11	19.01
FA 7.5% Concrete with quantity 5% of fly ash	1812.15	193.55	351.61	28.51
FA 10% Concrete with quantity 10% of fly ash	1812.15	193.55	342.11	38.01
FA12. 5% Concrete with quantity 5% of fly ash	1812.15	193.55	332.60	47.52
FA 15% Concrete with quantity 15% of fly ash	1812.15	193.55	323.10	57.02
FA 17.5% Concrete with quantity 5% of fly ash	1812.15	193.55	313.60	66.52
FA 20 % Concrete with quantity 20% of fly ash	1812.15	193.55	308.10	72.02

**Table 5:** Pre-determined natural frequencies of the ordinary concrete cube with the 20% addition of fly ash from sewage sludge ash.

Ordinary Concrete Cube with 20% Addition of Fly Ash from Sewage Sludge		Mode 1	Mode 2	Mode 3
		2119.214	2336.438	3094.247
Mode 1	2119.214	100	27.697	61.491
Mode 2	2336.438	27.697	100	1.322
Mode 3	3094.247	61.491	1.322	100

**Table 6:** Determined natural frequencies of the ordinary concrete cube with the 20% addition of fly ash from sewage sludge ash.

Ordinary Concrete Cube with 20% Addition of Fly Ash from Sewage Sludge		Mod	Mod
		2336.438	3094.247
Mode 2	2336.438	100	1.464
Mode 3	3094.247	1.464	100

## Data Availability

Data are not publicly available. The data may be made available upon request from the corresponding author.
